# Symbolic Analysis Applied to the Specification of Spatial Trends and Spatial Dependence

**DOI:** 10.3390/e22040466

**Published:** 2020-04-20

**Authors:** Maryna Makeienko

**Affiliations:** Economia Aplicada y Estadistica, International Doctorate in Economics, Universidad Nacional de Educacion a Distancia, 28015 Madrid, Spain; makeenkomari@gmail.com

**Keywords:** symbolic entropy, deterministic trends, specification

## Abstract

This article provides symbolic analysis tools for specifying spatial econometric models. It firstly considers testing spatial dependence in the presence of potential leading deterministic spatial components (similar to time-series tests for unit roots in the presence of temporal drift and/or time-trend) and secondly considers how to econometrically model spatial economic relations that might contain unobserved spatial structure of unknown form. Hypothesis testing is conducted with a *symbolic-entropy* based non-parametric statistical procedure, recently proposed by Garcia-Cordoba, Matilla-Garcia, and Ruiz (2019), which does not rely on prior weight matrices assumptions. It is shown that the use of geographically restricted semiparametric spatial models is a promising modeling strategy for cross-sectional datasets that are compatible with some types of spatial dependence. The results state that models that merely incorporate space coordinates might be sufficient to capture space dependence. Hedonic models for Baltimore, Boston, and Toledo housing prices datasets are revisited, studied (with the new proposed procedures), and compared with standard spatial econometric methodologies.

## 1. Introduction

Spatial trends have not played a prominent role in explaining and understanding how the outcomes in one geographical location are related to the outcomes in nearby locations (regions, countries, or points in space). This is especially evident in the spatial econometric literature. This absence contrasts with the role that time trends have played in time series econometrics for explaining economic outcomes that are close in temporal terms. One plausible explanation for this is that there are not enough statistical tools for testing if the data are compatible with a spatial trend. This paper aims to deal with the use of spatial trends when modeling and studying economic relations where directly or indirectly there are spatially correlated missing variables.

As one might think, questions regarding, e.g., how neighboring outcomes affect (or are affected by) the outcome (or behavior) of a given firm, state, county or—in general terms—a given institution, are central questions that are of interest in many scientific domains. Particularly, spatial spillovers and geographically clustered errors have been at the core of the development of spatial econometrics. For example, for real-estate economics, it is of much relevance to understand how neighboring home prices affect the sale price of a given house. It is not evident how traditional econometrics can deal with this spillover effect, while spatial econometrics [1,2] has proposed a well-known method to capture outcomes that might depend on outcomes in “nearby” locations but not those further away. To do so, a simple way to capture these restrictions is to define a *W* matrix to reflect spatial connectivity among neighbors, so-called spatial weight matrix, that have served as a basis for different econometric model specifications that explicitly incorporates spatial lags. A typical example, although there are many others that we comment and use in this paper, is the model Y=βX+ρWY+ε where *Y* is the dependent variable, *X* contains explanatory variables of *Y*, ε stands for the error term, and *W* is the weight matrix or matrix of connections. Another classic model is Y=βX+u, where u=ρWε+ν, i.e., there is spatial autocorrelation in the errors. There are other spatial econometric models that are used below that share a common denominator, namely that space enters into the equation through *W*. However, in this paper, we explore the possibility of introducing space differently (see [3,4]) whenever specification tests indicate it. From this point of view, one of the contributions of the paper is to build the steps between parametric spatial models and semiparametric ones. One of the key elements in this link is the role played by the weight matrix.

The use of the weight matrix *W* has been a controversial issue over the past few years. The two main and more severe critiques were by [5,6]. McMillen’s critique is based on the fact that introducing space via the error or the dependent variable is to control for unknown sources of spatial dependence, while most of the literature takes a leap on faith by considering or viewing the model as a correct parametric form. Furthermore, as argued by Gibbons and Overman, the use of *W* in the dependent variable and/or in the error structure might be pointless for identifying causal links and it also could be easily biased because *W* might be endogenous. Another related source of problems is that the specification of *W* is often arbitrary, as this selection is made a priori, depending on the user’s judgment. This decision is extremely important because, if matrix *W* is misspecified in some way, parameter estimates are likely to be biased and they will be inconsistent in models that contain some spatial lag, as stated in [7]. Furthermore, the consequences for evaluating effects of policy decisions can be serious if model specification is not conducted properly.

In this paper, we propose to statistically study other ways of incorporating space to control for unknown sources of spatial dependence before relying on *W*. We firstly focus on testing for (weak) spatial dependence in the presence of a leading deterministic components, similar to time-series tests for unit roots in the presence of drift and/or trend. To do so, we rely on a recent statistical procedure based on symbolic entropy developed in [8] [GMR] to determine whether a cross-sectional dataset is statistically compatible with a leading deterministic component in the form of a spatial trend. Secondly, we focus on well-known housing price datasets, and we study if some spatial trend can capture the spatial dependence. Thirdly, for those cases that were found statistically compatible with a spatial trends, a geographically restricted semiparametric approach is proposed to specify a model avoiding the critical points on *W*.

Some geographers and econometricians have highlighted the possibility of existence of spatial trends, and its application in the spatial analysis. The aim of trend analysis is to divide the spatial behavior of the data into (at least) two parts, namely the trend surface and the residuals, which are deviations of the measurements from the fitted trend surface, and they are the uncertain (random, stochastic) component. Caragliu and Nijkamp in [9] tried to identify spatial trends using Moran’s *I* statistics in their work as well. Basile and Ciccarelli in [10] studied a spatial location patterns of the various branches of the Italian manufacturing industry, including a simple semiparametric model with a smooth spatial trend to control for time-invariant unobserved heterogeneity. Earlier, Mur and Trivez [11] discussed the unit roots in spatial econometrics, introducing a common scale factor for all observations into the SAR model. Nevertheless, all the analyses performed were based on the assumption that the spatial trend already exists in the data. There existed no tools to test if this assumption is true on the raw data (or even in the residuals) before introducing trend models into the analysis. This procedure is somehow a criticizable method for model specification which is probably explained by the absence of available tests for spatial trends (authors relied on Moran’s or Geary’s tests that are well-established tests but for null hypothesis that are not related with trends). As indicated above, we use the GMR test for deterministic dynamics in spatial processes for specifying models. As a first step, we try to detect whether there exists a trend in a given spatial dataset, that is to say, we wonder if the observed data are compatible with spatial dependence in the form of a spatial trend, and, thus, we wonder how to model it. Modeling a spatial trend (if there is one) in our data might let us control for the variability of the phenomena occurred over the regions and therefore most of the problems that arose as a consequence of using and selecting *W* would not apply.

From an applied point of view, we focus our attention on the house market that has been already widely explored by spatial econometricians, among others. One of the reasons to incorporate spatial effects into the hedonic house price model is that the model form is intended to capture either interaction effects, market heterogeneity, or both. Another reason is that, here, spatial autocorrelation in omitted variables, or unobserved externalities and heterogeneities, are relegated to the error term [1,12].

It is realistic to consider that there might exist some type of spatial trend in hedonic housing models that aim to explain price on basis of their characteristics, among which geographical location is an important explanatory variable for price formation [12]. To make a proper analysis, we consider three datasets on house pricing and along with the characteristics of the houses sited in Baltimore, Boston, and Toledo to estimate several hedonic models implementing the proposed approach. We aim at answering if spatial trends (linear and/or nonlinear) together with other explanatory variables (house characteristics) are able to properly capture the spatial structure for these three datasets. All the models in this paper were estimated with Matlab and R routines that are available for interested readers. The same availability applies to all datasets.

The remainder of the paper is organized as follows. Section 2 describes the methodology that is applied in Section 3, where we describe the analysis and the results obtained, and elaborate on comparisons obtained with other available methods. Section 4 concludes and provides some hints for further research on the topic. An extensive appendix is supplied to complete all models’ estimations because otherwise reading the paper would be less effective.

## 2. Methodology

As mentioned in the Introduction, central to the paper is the use of a test for spatial deterministic structure, which is based in symbolic analysis. Symbolic analysis has been previously used in the geographical literature to (help) specify spatial models. The interested reader is referred to the works of [13,14,15]. Recently, GMR, using symbolic methods, is put forward as a nonparametric statistical procedure for testing for misspecification in terms of potential spatial trends. According to the authors, the proposed nonparametric statistic—which we refer to as delta-test—is aimed to cover a clear and well-known gap in the literature, namely to test if raw spatial data are compatible with some form of spatial trend.

The most used nonparametric test for spatial dependence is the Moran’s *I* test, which aims to test if there exists a spatial dependence in the data or not. Moran’s *I* is a correlation coefficient that measures the overall spatial autocorrelation of the dataset. It measures how one object is similar to others surrounding it. If objects are attracted by each other, it means that the observations are not independent. Despite the fact that Moran’s test is designed for testing spatial structure, it is unable to detect certain spatial nonlinear dependences. For example, consider the spatial model $X_{s}=aX_{s-1}\left(1-X_{s-1}\right)$ with fixed initial conditions ($X_{0}=0.7,a=3.57)$, where the set of neighbors of *s* is {s+1,s+2,…,s+m+1}, m,s∈N, has an obvious and simple spatial structure, which also lacks of stochastic components. However, Moran’s I test fails to reject the null of no spatial autocorrelation, regardless the sample size. One plausible explanation for this performance is that Moran’s test is not designed to deal with nonlinearities. Fortunately, delta-test is robust against nonlinearities of simple and complex form and therefore is able to clearly capture the spatial trend, as have been shown in GMR.

A generally accepted practice has been to test for spatial dependence using Moran’s test on georeferrenced economic raw data. If the test rejects the null of no spatial dependence, the data analyst might consider several forms of building spatial econometric models. The vast majority of researchers will try to specify and then estimate a model of the general type

$$Y=\rho WY+\alpha \iota_{n}+X\beta +WX\theta +u,u=\lambda Wu+\varepsilon$$

where *Y* represents an n×1 vector consisting of one observation on the dependent variable for every unit in the sample (i=1,…,n), $\iota_{n}$ is an n×1 vector of ones associated with the constant term parameter α, *X* denotes an n×k matrix of explanatory variables associated with the k×1 parameter vector β, and $\varepsilon ={\left(\varepsilon_{1},\ldots ,\varepsilon_{n}\right)}^{T}$ is a vector of independently and identically distributed disturbance terms with zero mean and variance $\sigma^{2}$. As mentioned in the Introduction, this has been criticized mainly for the problems derived from incorporating space dependence with W. Notice that all these specifications consider that the potential spatial model has no deterministic spatial trends. This in part is probably due to the fact that until now there has been no way to test for leading spatial trends.

The delta-test, which we briefly describe below, tests for the null of a non-stochastic leading term in a spatial dataset ${\left\{X_{s}\right\}}_{s\in S}$ where *S* is a set of coordinates. To do so, the spatial realization ${\left\{X_{s}\right\}}_{s\in S}$ is embedded in an *m*-dimensional space:

$$X_{m}\left(s_{0}\right)=\left(X_{s_{0}},X_{s_{1}},\ldots ,X_{s_{m-1}}\right)\mathrm{for}s_{0}\in S$$

where $N_{s}=\left\{s_{1},\ldots s_{m-1}\right\}$ are the m−1 nearest neighbors to $s_{0}$. A symbolization map is then defined $f:{\left\{X_{s}\right\}}_{s\in S}↪R^{m}\to \Gamma$ as:

(1) $$f\left(X_{s}\right)=\left(I_{ss_{1}},I_{ss_{2}},\ldots ,I_{ss_{m-1}}\right)$$

where $I_{ss_{j}}$ is an agreement indicator function of being above or below the median at locations *s* and $s_{j}$. Γ is the set of $2^{m-1}$ different vectors of dimension m−1 with entries in the set {0,1}, where we refer to each symbol by $\sigma_{i}$. Obviously, it is required that the spatial process $X_{s}$ has a finite median, otherwise the test cannot be applied, which is not a very strict limitation. Then, the relative frequency, $p_{\sigma }$, of each symbol is computed from the data, and the associated entropy of the dataset is calculated: $h\left(\Gamma \right)=-\sum_{\sigma \in \Gamma }p_{\sigma }\ln\left(p_{\sigma }\right)$. The delta-test consists on estimating the behavior of a function of the difference between entropies $h^{W_{j+1}}\left(\Gamma \right)-h^{W_{j}}\left(\Gamma \right)$ where $W_{j}$ and $W_{j+1}$ are sets of symbols chosen at random from Γ. Under the null of a non-stochastic spatial structure, that difference does not increases with the number of considered symbols.

Particularly, the delta-test is implemented by testing if $\alpha_{1}=0$ in the following regression

(2) $$dh^{W_{j}}\left(\Gamma \right)=\alpha_{0}+\alpha_{1}j+\varepsilon_{j},\;\mathrm{for}\;j=1,2,\ldots k-1$$

where

$$dh^{W_{j}}\left(\Gamma \right)=\frac{h^{W_{j+1}}\left(\Gamma \right)-h^{W_{j}}\left(\Gamma \right)}{\log\frac{j+1}{j}}.$$

The delta statistic is a test well-suited to detect simple and complex spatial trends. Provided with the delta-test, (dh−test) in the following tables, we can supplement the spatial analysis by firstly applying the test to the spatial raw data. In the case of a non-rejection of the null hypothesis of deterministic spatial leading term, the possibility of specification of a scenario with spatial (deterministic) trends opens for the econometric modeler. A natural way for modeling this situation from an econometric point of view is by using what we call restricted semiparametric regression:

(3) $$Y=\alpha \iota_{n}+X\beta +f\left(a,b\right)+\varepsilon$$

where each element on vector *Y* is a continuous output variable in a given location. Xβ contains all explanatory variables (i.e., a set of explanatory variables that can include categorical variables and where vector β collects fixed parameters). The important nonparametric part f(a,b) is restricted to geographic functions of longitude and latitude, a,b, respectively. At this point, according to the rejection of the null hypothesis of non-stochastic spatial leading term, there is no evidence for introducing in the model a weight matrix (*W*), neither in the parametric part Xβ nor in the nonparametric one.

Several comments are important thereon. (i) The previous family of models aims to ascertain whether a specification of space via latitude and longitude might serve to control for spatial dependence, once the researcher has had statistical evidence of a spatial trend. (ii) At this stage, prior to the use of a given *W* weight matrix, we wonder if considering some form of geographical variables in the model is enough to correctly estimate vector β. This will avoid the severe consequences in estimation and inference (about β) of not considering spatial dependence when it really exists, as occurs in many fields. (iii) Notice also that the family of models in Equation (3) will not be an object of the main critiques that spatial econometrics has received by scholars, that we comment in in the previous section.

The delta-test can be used as a diagnostic tool helping in the model selection procedure. Consider a model that erroneously omits some form of spatial dependence

$$Y=\alpha \iota_{n}+X\beta +u;$$

we understand that the omission can be in the form of a linear spatial dependence or in the form of a spatial trend. An example of the former is

u:=WXθ+ε,

while the latter can be of the form

u:=μf(a,b)+ε.

However, the choice between these specifications is non-trivial. Interestingly, for model specification, the delta-test can be used to distinguish between them if the test is applied to the residuals of the misspecified model, that is, if it is applied to $\hat{u}$. In the case of a true spatial dependence via *W*, the delta-test will tend to point that no spatial trend is found in the residuals, and therefore the researcher will have to deal with a statistically correct specification of the model (this will be probably done through well-known models in the spatial econometrics literature, as we indicate below in this paper). In this regard, we expect that Moran’s *I* test will correctly indicate spatial autocorrelation in the residuals. On the contrary, the delta-test will point out that a spatial trend is omitted if the true spatial dependence comes in the form a non-stochastic geographic spatial structure (spatial trend). Obviously, the researcher should now take a different modeling strategy, as she has put forward a statistically compatible spatial trend. In other words, it should be required to propose some form of f(a,b).

Our proposed selection procedure consists on specifying the model using the previous diagnostics’ tools. Particularly, we firstly run delta-test on the raw data to check for existence of deterministic structure and we also run Moran’s test to check if there is a spatial autocorrelation in the data we use. If delta-test cannot reject the statistical existence of a spatial trend, we introduce a geographical additive model of the form given in Equation (3). In particular, we consider and study two forms for the geographically restricted nonparametric part, f(a,b). The first way (that we refer to it as delta-model strategy) is to restrict f(a,b) to be a low-degree polynomials of coordinates, which is inspired in the practice in time-series modeling of including powers of *t*-time.

- $f_{A}\left(a,b\right)=a+b+a^{2}+b^{2}+ab$
- $f_{B}\left(a,b\right)=a+b+a^{2}+b^{2}$
- $f_{C}\left(a,b\right)=a+b+ab$
- $f_{D}\left(a,b\right)=a^{2}+b^{2}+ab$
- $f_{E}\left(a,b\right)=a^{2}+b^{2}$
- $f_{F}\left(a,b\right)=a+b$
- $f_{G}\left(a,b\right)=a^{3}+b^{3}$

We use letters A,B,…,G to indicate the model specification we refer to. For example, by Model B, we mean $Y=\alpha \iota_{n}+X\beta +f_{B}\left(a,b\right)+\varepsilon$. The second way of geographically restricting the general term f(a,b) consists on modeling according to

(4) $$Y=\alpha \iota_{n}+X\beta +f\left(z\left(a,b\right)\right)+\varepsilon$$

where vector *z* is a set of variables whose effects enter the equation non-parametrically. In this paper, we consider the following configurations of the nonparametric part:

(5) Spline:f(z)=(a,b)

where f(z) is fully nonparametric and is limited to variables longitude and latitude;

(6) $$C-spline:f\left(z\right)=\beta_{0}+\beta_{1}\left(x-x_{0}\right)+\beta_{2}{\left(x-x_{0}\right)}^{2}+\beta_{3}{\left(x-x_{0}\right)}^{3}+\sum_{s=1}^{S}\delta_{s}{\left(x-x_{s}\right)}^{3}D_{s}$$

where the spline simply adds a set of interaction terms between dummy variables and cubic terms to a standard cubic function, and where S are equal intervals ranging from $x_{0}=min\left(x\right)$ to $x_{S}=max\left(x\right)$ and there is a dummy variable $D_{s}$ indicating whether *x* is greater than $x_{s}$. C-spline is used as an analogy approximation to the G-model we introduced before. This allows us to make better comparison of the models that are in the same analysis line. Finally, a Fourier based spline of the form

(7) $$F-spline:f\left(z\right)=\beta_{0}+\beta_{1}z+\beta_{2}z^{2}+\sum_{j=1}^{J}\left(\gamma_{j}sin\left(jz\right)+\lambda_{j}cos\left(jz\right)\right)$$

where z=2π(x−min(x))/(max(x)−min(x)).

Recall that splines and series regression are based on the mathematical theory of the approximation of functions. Particularly, spatial-econometricians that are concerned with approximating the conditional expectation function find very useful the Weierstrass–Stone theorem A.1 which states that any continuous function can be uniformly well approximated by a polynomial of sufficiently high order, under mild regularity conditions. There are mathematical results that point out that, when the true conditional expectation function is smoother, it is possible to approximate it with a fewer number of series terms. This explains why other spline methods such as B-splines or P-Splines can be used instead of (or together with) the ones we have selected. The central point is the same one as in the delta-models: basically considering coordinates can be a first step to control for spatial relationships. One or more of these simple structures (i.e., a family of models) can approximate a spatial trend even in the case of a nonlinear spatial trend.

Potentially, all these geo additive models can therefore be used to model the initial detected spatial trend on the raw data. Once estimated all these models, we choose the estimated one that has residuals that indicate absence of spatial dependence. To this end, delta and Moran’s tests are both used in the residuals of each model.

Alternatively, if the delta-test on the raw data points to no spatial structure, while Moran’s I does, then spatial models of the form indicated in Table 1 have to be used. Once these models have been estimated, there will be some models for which Moran’s I (and also delta-test) indicate that no spatial structure is remaining in the residuals. In case there is more than one estimated model, then we use AIC criterion for choosing one of them.

The selection criteria and an illustrative form for how to choose the model are given in Figure A1. In the next section, the proposed specification procedure is illustrated on three real spatial datasets.

## 3. Modeling Housing Prices via Hedonic Models

Numerous economists have empirically considered that the price of a house, building or piece of land is determined by the characteristics of the property itself (e.g., its size, appearance, features such as patios or rooms or fireplaces, and condition of the property), as well as characteristics of its surrounding environment (e.g., if the neighborhood has a high crime rate and/or is accessible to schools and a downtown area, the level of water and air pollution, or the value of other homes close by). To model the relation between price and those characteristics, several hedonic models have been used. Hedonic pricing is a model which identifies price factors according to the premise that price is determined both by internal characteristics of the good being sold and external factors affecting it. From our point of view, the relevance of estimating a hedonic price models is to estimate the extent to which each factor (characteristic) affects the price of the home. Apart from classic hedonic models, [16] proposed a semiparametric approach, including a $f(x_{c},y_{c})$ nonparametric function of the location of the observations. Day, et al. [17] used a two-dimensional vector of coordinates to explain the variability in housing prices. Additionally, [18] used the coordinates of localization (latitude and longitude) of each observation as heteroskedasticity source in their analysis. Although there exist studies that tried to include the location as an explanatory variable, there were no existing tool that could permit us make an analysis without including W. Thus, in our analysis, we check if there might exist a model that merely incorporating space coordinates might be sufficient to capture space dependence. For this reason, we focus our study on well-studied data on housing prices in Baltimore (https://geodacenter.github.io/data-and-lab//baltim/), Boston (https://geodacenter.github.io/data-and-lab//boston-housing/), and Toledo (https://www.spatial-econometrics.com/), as these are perfect examples of the market where space, along with other characteristics, is directly introduced. Over the past years, researchers have made a great input to the housing prices analysis, taking into account spatial and temporal factors. Thus, we complement the existing analysis, including the possibility of spatial trends existence in the data.

### 3.1. Baltimore Housing Prices

House prices and characteristics from Baltimore were firstly studied by [19]. The data come from the 1978 multiple listings for Baltimore (Maryland) that contain structural descriptors of the house, the sales price, and the address. Each house is assigned coordinates by locating the address on the Maryland coordinate system. The dependent variable is the selling price of the house and the exogenous variables include the attributes of the structure of the house and its sale: Number of rooms (NROOM), information on if the house is a detached unit (=1) or not (DWELL), number of bathrooms (NBATH), if the house has patio (PATIO), fireplace (FIREPL), air conditioning (AC), basement (BMENT), number of stories (NSTOR), number of car spaces in garage (GARAGE), age of dwelling (AGE), dummy variable that takes a value of 1 if the dwelling is located in Baltimore county (CITCOU), lot size (LOTSZ), interior living area (SQFT), and the month of 1978 in which house was sold (STIME).

Moran’s test on the data on Baltimore housing prices gives a clear evidence of the spatial autocorrelation (Table 2). The delta-test on the raw data confirms the presence of deterministic structure, which gives evidence in favor of running restricted semiparametric analysis, including spatial trend. Following the modeling proposal given in the previous section (see also Figure A1), we firstly model the deterministic part by using the so-called delta-models. Results for Models A and B are clearly in favor, as the estimated model controls for the spatial dependence of the data: both delta-test and Moran’s test indicate that spatial structure is controlled with Models A and B. In other words, our estimation of the extent to which each characteristic of the house affects the house’s price is safely estimated, which was one of the main concerns as initially claimed. The same conclusion is reached if we opt for some spline-based methods.

For illustrative purposes, we show what happens if instead we model according to models in Table 1, despite the fact that delta test results do not recommend to take that modeling strategy. To this end, we estimate all the W-based model specifications and we find that the best spatial models for our data are SDM and SEM models. This conclusion is reached by using AIC. One interesting conclusion of the results given in Table 2, is that using ACI criteria and neglecting the use of delta-test, will lead us to a improvable specification as neither SDM nor SEM models are able, according to delta-test results, to remove the previously found spatial trend. In other words, the residuals of these models are compatible with a deterministic structure that has not yet been removed. For this reason, results seem to point out that restricted semiparametric models work better in this case, as they let us get rid of the spatial structure of the model and thus get more credible results on the estimates. The practical implications for Baltimore housing prices are mainly relative to the partial effects of several explanatory variables, but not to the list of significant variables or to the signs.

### 3.2. Boston Housing Prices

The second dataset includes data for census tracts in the Boston Standard Metropolitan Statistical Area (SMSA) in 1970. These data were used by [20]. It is interesting mainly because it considers environmental characteristics as hedonic determinants of the price. The Boston sample contains 506 census tracts (one observation per census tract) on 14 non-constant independent variables (with tracts containing no housing units or comprised entirely of institutions excluded). These variables include crime rate (CRIME), proportion of area zoned with large lots (ZONING), proportion of non-retail business area (INDUSTRY), location contiguous to the Charles River (CHARLESR), levels of nitrogen oxides (NOXSQ), average number of rooms (ROOMS), proportion of structures built before 1940 (HOUSEAGE), weighted distances to the employment centers (DISTANCE), an index of accessibility (ACCESS), property tax rate (TAXRATE), pupil–teacher ratio (PUPIL/TEACHER), black population proportion (BLACKPOP), and lower status population proportion (LOWCLASS).

The results of Moran’s test and delta-test on the data on Boston housing prices give a clear evidence in favor of spatial structure. After estimating spatial trends, the results (Table 3) show that the deterministic part has been removed, but there still is a spatial structure according to Moran’s *I*.

Therefore, it is justified modeling according to models given in Table 1. All the results are reported in Table A2 in appendix. In this case, the best choice would be GNS and SAC models that correct for spatial structure in the sense that both Moran and delta-tests statistically indicate that spatial structure has been controlled (see Table 3). AIC values on these two models suggest choosing SAC, but the difference is very mild. As happened with the previous dataset, the list of relevant explanatory variables is common to all the models. Variations are again on the partial effects.

### 3.3. Toledo Housing Prices

The third dataset is the dataset on housing prices in Toledo, Ohio, United States. It includes data on average house values for 98 census tracts along with 10 explanatory variables and latitude–longitude coordinates. Explanatory variables include the neighborhood quality (Neighborhood), net lot square foot (Lot sqft), total square foot living area (Total sqft), family room conditions (Family room), recreation room conditions (Rec room), air conditioning (Air cond), number of bathrooms (Baths), condition of the house (Condition), garage condition (Garage condition), and the age of the house (Age).

As happened with the other two datasets, Moran’s test and delta-test report spatial structure. As regards restricted semiparametric models, it is worth mentioning that both delta models and spline models are able to control for both types of spatial dependences, as can be observed in Table 4, which can be completed with information found in the appendix. This is similar to what happened with Baltimore’s housing dataset and model. The interesting difference with respect to Toledo’s model is that there is also a spatial model (from the set of models given in Table 1) that also controls for spatial dependence, namely SEM estimated model. Now, the researcher has to choose between modeling with a geo additive model or with a W based model. In both cases, the estimated models are able to control for spatial dependence. If the modeler is uncomfortable with or suspicious of using a given W matrix because of some of the reasons explained in the Introduction of the paper, we would recommend using a restricted semiparametric model approach. From the practical point of view, it is remarkable that for this dataset the variable “condition” has no statistical relevance if semiparametric models are adopted, while it has an impact if the SEM model is selected. Something similar happens with the fact that the house does or does not have a recreation room.

## 4. Conclusions

The focus in this paper is on cross-sectional spatial data modelization. The article contributes, on the one hand, to the use of specification tests in order to assess the robustness of the results, once it is recognized that our ability to accurately model spatial data is very limited. On the other hand, it also contributes to the debate around the W weight matrix in formulating spatial econometrics models. There are several challenges when econometrically modeling economic relations for which space can have a relevant role. The combination of missing correlated over space variables along with many sources of potential nonlinearities has led scholars to consider that conventional parametric spatial models are not necessarily the best modeling strategy. We firstly argue that it is critical to use specifications tests in order to validate results. This explains why we propose (and develop on): (i) the use of a nonparametric test for spatial structure of unknown (either linear o nonlinear) form along with other available tests; and (ii) the use of geographically-restricted semiparametric models that assume that the true model is unknown. To put it differently, we argue that, supported by powerful statistical tools, semiparametric models and estimators could be used before going through conventional spatial lag models.

This article has studied hedonic price functions and estimates because housing price formation has been a central object for the implementation of policy decisions and therefore model specification may be particularly important. The three studied cites are independent among them. Regarding specifications issues, the main conclusion is the following: spatial dependence structure (potentially of unknown form) can be controlled by restricted semiparametric models that do not use W matrix specification; however, there might be spatial data relationships for which spatial structure can only be controlled at the cost of assuming a W matrix of connections. Given the potential consequences of a wrong specification, those models that avoid a W matrix are preferred. However, if the modeler requires the use of weight matrix (as might happen), according to our results, robust specification tests are recommendable to be used to choose the best parametric model.

Several lines of further research can be proposed. From a boarder point of view, this paper contributes to the spatial econometric literature around open concerns for the scholars, in particular to the literature on spatial dependence bias, on functional form bias and on spatial dependence bias. However, other concerns remain open for further research, namely the potential interaction between geo additive terms and covariates of particular interest for researchers. Moreover, it might be the case that the proposed strategy could be compatible with the models that include other sources of spatial autocorrelation, which can be studied further on. From the applied side, this study centered on housing prices; however, there are other well studied cross-sectional datasets (along with their economic relationships) for which space is a natural source of variation and explanation that has kept the attention of geographical modelers (industry location and knowledge spillovers, among others). In this vein, some models could be revised to double-check and understand the existence of remaining traces of spatial dependence.

## Figures and Tables

**Table 1:** Spatial model specification.

GNS	$Y=\rho WY+\alpha \iota_{n}+X\beta +WX\theta +u$
	u=λWu+ε
SAC	$Y=\rho WY+\alpha \iota_{n}+X\beta +u$
	u=λWu+ε
SDM	$Y=\rho WY+\alpha \iota_{n}+X\beta +WX\theta +\varepsilon$
SDEM	$Y=\alpha \iota_{n}+X\beta +WX\theta +u$
	u=λWu+ε
SAR	$Y=\rho WY+\alpha \iota_{n}+X\beta +\varepsilon$
SLX	$Y=\alpha \iota_{n}+X\beta +WX\theta +\varepsilon$
SEM	$Y=\alpha \iota_{n}+X\beta +u$
	u=λWu+ε
	if θ=−ρβ, then λ=ρ
OLS	$Y=\alpha \iota_{n}+X\beta +\varepsilon$

**Table 2:** Model comparison for Baltimore housing prices.

	OLS	Model A	Model B	SEM	SDM	C-Spline	F-Spline
Constant	7.16	2799.08	2864.02 *	6.95	13.05	−17,690.00	−48.30
Nroom	0.29	0.21	0.21	0.75	0.29	0.33	0.29
Dwell	6.30 **	6.09 **	6.12 **	6.96 ***	6.35 ***	6.79 **	6.95 **
Nbath	6.08 ***	5.71 ***	5.75 ***	6.57 ***	5.59 ***	5.82 **	5.89 **
Patio	9.40 ***	7.94 ***	7.98 ***	7.55 ***	5.21 ***	8.16 **	7.94 **
Firepl	10.69 ***	8.92 ***	8.95 ***	8.95 ***	8.88 ***	8.80 ***	8.66 ***
AC	8.20 ***	6.26 ***	6.25 ***	7.34 ***	7.17 ***	6.91 **	6.93 **
Bment	3.81 ***	3.71 ***	3.69 ***	3.59 ***	3.36 ***	3.36 **	3.41 ***
Nstor	−4.36	−4.63 *	−4.60 *	−3.93	−2.44	−4.19	−4.08
Gar	5.53 ***	5.15 ***	5.14 ***	5.23 ***	5.74 ***	4.93 **	4.94 **
Age	0.00	0.01	0.01	−0.01	0.00	−0.02	−0.03
Citcou	12.46 ***	10.72 ***	10.74 ***	12.43 ***	10.86 ***	16.55 ***	16.83 ***
Lotsz	0.04 **	0.03 *	0.03 *	0.03	0.02	0.04 *	0.04 *
Sqft	0.37 *	0.31	0.31	0.26	0.25	0.29	0.28
Moran (*p*-value)	0.02	**0.25**	**0.29**	0.02	0.46	**0.32**	**0.34**
dh-test (*p*-value)	0.02	**0.02**	**0.02**	0.12	0.12	**0.02**	**0.01**
AIC				1.53	1.51		

#of embedding dimensions m = 6; dh-test *p*-value on raw data 0.13; ***, **, * = coefficient estimates that are significant at the 0.01, 0.05 and 0.1 level, respectively. Numbers in bold state if the model controls for spatial structure (Moran *p*-value > 0.1) and/or controls the spatial trend in the data (dh-test *p*-value < 0.1).

**Table 3:** Model comparison for Boston housing prices.

	OLS	Model A	Model B	SAC	GNS	C-Spline	F-Spline
Constant	36.46 ***	46.69 ***	46.78 ***	28.09 ***	25.74 ***	704,400.00 **	−274.30
Crime	−0.11 ***	−0.13 ***	−0.13 ***	−0.13 ***	−0.15 ***	−0.12 ***	−0.12 ***
Zoning	0.05 ***	0.03 **	0.03 **	0.04 ***	0.03 **	0.04 **	0.04 **
Industry	0.02	0.02	0.02	−0.01	−0.02	0.03	0.03
Charlesr	0.02 ***	2.68 ***	2.69 ***	−0.27	−0.1	2.42 **	2.15 *
Noxsq	−17.77 ***	−21.80 ***	−21.83 ***	−17.80 ***	−19.23 ***	−20.18 ***	−19.33 ***
Rooms	3.81 ***	3.74 ***	3.74 ***	4.24 ***	4.66 ***	3.62 ***	3.58 ***
Houseage	0.01 ***	−0.02	−0.02	−0.02	−0.03 **	−0.02	−0.02
Distance	−1.48 ***	−3.22 ***	−3.24 ***	−1.62 ***	−1.61 ***	−2.37 ***	−2.45 ***
Access	0.31 ***	0.36 ***	0.36 ***	0.32 ***	0.31 ***	0.36 ***	0.35 ***
Taxrate	−0.01 ***	−0.01 ***	−0.01 ***	−0.01 ***	−0.01 ***	−0.01 ***	−0.01 ***
Pupil/Teacher	−0.95 ***	−1.02 ***	−1.02 ***	−0.64 ***	−0.57 ***	−0.99 ***	−0.95 ***
Blackpop	0.01 ***	0.01 ***	0.01 ***	0.01 ***	0.01 ***	0.01 ***	0.01 ***
Lowclass	−0.52 ***	−0.53 ***	−0.54 ***	−0.43 ***	−0.39 ***	−0.53 ***	−0.53 ***
Moran (*p*-value)	0	0	0	**0.46**	**0.54**	0	0
dh-test (*p*-value)	0.04	0.03	0.03	**0.01**	**0.01**	0.03	0.03
AIC				2.89	2.88		

#of embedding dimensions m = 7; dh-test *p*-value on raw data 0.20; ***, **, * = coefficient estimates that are significant at the 0.01, 0.05 and 0.1 level, respectively. Numbers in bold state if the model controls for spatial structure (Moran *p*-value > 0.1) and/or controls the spatial trend in the data (dh-test *p*-value < 0.1).

**Table 4:** Model comparison for Toledo housing prices.

	Model A	Model G	SEM	SDM	C-Spline	F-Spline
Constant	1,252,522,880	1,224,987.63 **	8410.68	−4299.56	3.299 $\times \;10^{8}$	3.445 $\times \;10^{6}$ *
Neighborhood	−2165.26	−2377.64	−2821.70	−895.53	−1348.00	−1162.00
Lot sqft	0.56	0.38	0.29	0.11	0.55	0.54
Total sqft	2.67	4.78	7.15	5.05	3.95	3.86
Family room	25,736.93 ***	30,321.64 ***	30,546.77 ***	21,876.78 ***	29,010.00 **	28,620.00 **
Rec room	−5686.75 **	−2600.6 *	−1832.99	−4104.28	−4645.00	−5318.00
Air cond	−22,861.58	−34,788.76 **	−37,228.27 ***	−23,296.76	−27,840.00	−25,680.00
Baths	19,030.72 ***	17,723.07 ***	16,604.19 ***	18,497.00 ***	18,410.00 ***	18,650.00 ***
Condition	5442.27	7472.95	8190.45 *	8672.42 **	5434.00	4823.00
Garage condition	−9686.32 ***	−10,585.34 ***	−10,895.37 ***	−11,240.48 ***	−9691.00 ***	−9479.00 ***
Age	−261.17 **	−188.05 *	−144.95	−225.93 **	−257.00 *	−265.50 *
Moran (*p*-value)	**0.89**	**0.88**	**0.57**	0.43	**0.51**	**0.53**
dh-test (*p*-value)	**0.01**	**0.02**	**0.05**	0.11	**0.01**	**0.01**
AIC			1.93	1.93		

#of embedding dimensions m = 5; dh-test *p*-value on raw data 0.11; ***, **, * = coefficient estimates that are significant at the 0.01, 0.05 and 0.1 level, respectively. Numbers in bold state if the model controls for spatial structure (Moran *p*-value > 0.1) and/or controls the spatial trend in the data (dh-test *p*-value < 0.1).

**Figure A1:**
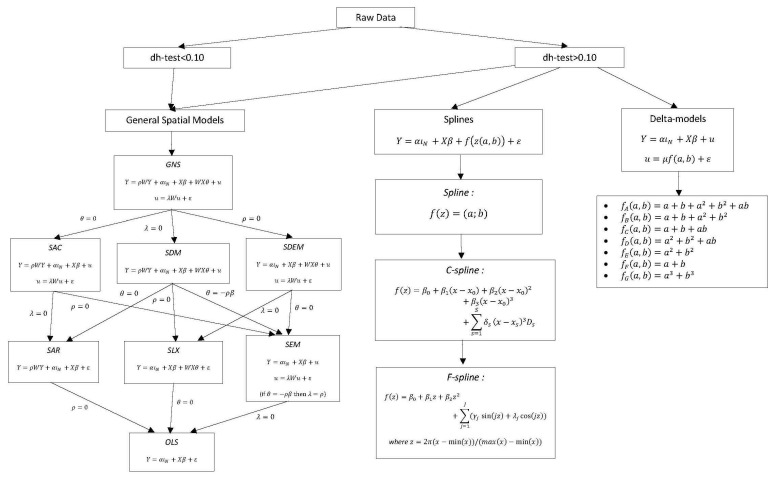
Model selection.

## Appendix A

### Appendix A.1. Weierstrass–Stone Theorem

If *X* is any compact space, let *A* be a subalgebra of the algebra C(X) over the reals R with binary operations + and ×. Then, if *A* contains the constant functions and separates the points of *X* (i.e., for any two distinct points *x* and *y* of *X*, there is some function *f* in *A* such that f(x)≠f(y)), *A* is dense in C(X) equipped with the uniform norm. This theorem is a generalization of the Weierstrass approximation theorem.

### Appendix A.2. Mechanism of Choosing the Best-Fit Spatial Model (Spatial Mechanism)

After running spatial models’ regressions, one of the criteria used to choose is the likelihood ratio (LR) test based on the log-likelihood function values of the different models. The LR test is based on minus two times the difference between the value of the log-likelihood function in the restricted model and the value of the log-likelihood function of the unrestricted model: $-2\times (logL_{restricted}-logL_{unrestricted})$. This test statistic has a Chi squared distribution $\chi_{n}^{2}$ with *n* degrees of freedom equal to the number of restrictions imposed. The election rule states that, if $LR_{test}>\chi_{n}^{2}$, then the unrestricted model performs better than the restricted one. Using this criterion, we can make a comparison of the models, the way shown below.

OLS vs. SLX

OLS vs. SAR

OLS vs. SEM

SAR vs. SAC

SEM vs. SAC

SLX vs. SDM

SEM vs. SDM

SAR vs. SDM

SLX vs. SDEM

SAR vs. SDEM

SEM vs. SDEM

Other models cannot be compared among themselves with LR test, as they are not nested. The only two models that are not nested but can be compared are SAR and SEM models. The criteria used to make a comparison is the Lagrange multiplier tests [21] for the SAR and SEM model specification tests, robust to the presence of other types of spatial interactions), which make it possible to choose among the SAR, SEM, or non-spatial model. The rest of the models can only be subjectively compared, checking the significance of the estimated coefficients and the structure of the model.

### Appendix A.3. Model Selection

Our procedure (Figure A1) consists in specifying the model using the previous diagnostics’ tools. Particularly, we first run delta-test on the raw data to check for existence of deterministic structure and Moran’s test to check if there is a spatial autocorrelation in the data we use. If delta-test cannot reject the statistical existence of a spatial trend, we introduce and estimate a geographical additive model of the form given in Equation (4). On the parallel line, we introduce and estimate spline models. After the estimation of all the models, we choose the one that controls both the spatial structure of the data and the spatial trend existence from each set of the model (geo additive and spline sets), evaluating the *p*-value of the Moran test and dh-test. Then, we repeat the same procedure for classic spatial models; if there is no model that controls both spatial structure and spatial trend presence, we choose the one that is the best following Akaike criterion.

**Table A1:** Results for Baltimore housing prices.

	OLS	SAR	SEM	SLX	SAC	SDM	SDEM	GNS	Spline	C-Spline	F-Spline	Model A	Model B	Model C	Model D	Model E	Model F	Model G
Constant	7.16	−0.91	6.95	8.78	−0.05	13.05	3.18	16.66 **		−17,690.00	−48.30	2799.08	2864.02 *	785.57	2.41	3.46	−3.84	5.98
Nroom	0.29	0.53	0.75	0.27	0.08	0.29	0.22	0.47	0.95	0.33	0.29	0.21	0.21	0.15	0.19	0.17	0.17	0.16
Dwell	6.30 **	5.74 **	6.96 ***	6.33 **	4.46 **	6.35 ***	7.52 ***	6.72 ***	7.13 ***	6.79 **	6.95 **	6.09 **	6.12 **	5.51 **	5.03 *	5.28 **	5.30 **	5.26 **
Nbath	6.08 ***	5.37 ***	6.57 ***	5.57 ***	4.08 **	5.59 ***	6.87 ***	6.72 ***	6.19 ***	5.82 **	5.89 **	5.71 ***	5.75 ***	5.67 ***	4.97 **	5.32 ***	5.34 ***	5.31 ***
Patio	9.40 ***	6.92 ***	7.55 ***	5.25 *	7.39 ***	5.21 ***	9.81 ***	5.63 **	6.34 **	8.16 **	7.94 **	7.94 ***	7.98 ***	9.35 ***	8.64 ***	8.98 ***	9.02 ***	8.94 ***
Firepl	10.69 ***	8.21 ***	8.95 ***	8.93 ***	8.46 ***	8.88 ***	9.48 ***	7.59 ***	7.58 ***	8.80 ***	8.66 ***	8.92 ***	8.95 ***	10.05 ***	9.55 ***	9.86 ***	9.89 ***	9.83 ***
AC	8.20 ***	7.15 ***	7.34 ***	7.18 ***	7.12 ***	7.17 ***	7.14 ***	7.25 ***	6.85 ***	6.91 **	6.93 **	6.26 ***	6.25 ***	6.29 **	6.50 ***	6.35 **	6.41 **	6.28 **
Bment	3.81 ***	3.78 ***	3.59 ***	3.36 ***	3.77 ***	3.36 ***	3.34 ***	3.02 ***	3.37 ***	3.36 **	3.41 ***	3.71 ***	3.69 ***	3.39 ***	3.74 ***	3.52 ***	3.53 ***	3.51 ***
Nstor	−4.36	−5.08 *	−3.93	−2.41	−5.82 **	−2.44	−1.20	−3.13	−3.49	−4.19	−4.08	−4.63 *	−4.60 *	−4.73	−5.23 *	−4.99 *	−4.98 *	−5.01 *
Gar	5.53 ***	5.65 ***	5.23 ***	5.74 ***	5.71 ***	5.74 ***	4.58 ***	4.86 ***	4.33 **	4.93 **	4.94 **	5.15 ***	5.14 ***	5.52 ***	5.76 ***	5.61 ***	5.62 ***	5.60 ***
Age	0.00	0.04	−0.01	0.01	0.06	0.00	0.03	0.01	0.01	−0.02	−0.03	0.01	0.01	0.01	0.01	0.01	0.01	0.01
Citcou	12.46 ***	10.12 ***	12.43 ***	10.87 ***	9.08 ***	10.86 ***	13.42 ***	10.32 ***	11.52 ***	16.55 ***	16.83 ***	10.72 ***	10.74 ***	13.10 ***	12.67 ***	13.18 ***	13.17 ***	13.18 ***
Lotsz	0.04 **	0.02	0.03	0.02	0.02	0.02	0.04 ***	0.01	0.03	0.04 *	0.04 *	0.03 *	0.03 *	0.03 **	0.03 *	0.03 *	0.03 *	0.03 *
Sqft	0.37 *	0.38 *	0.26	0.25	0.47 **	0.25	0.08	0.18	0.19	0.29	0.28	0.31	0.31	0.31	0.37	0.34	0.34	0.34
WNroom				0.02		0.02	−2.07 ***	−4.37 **										
WDwell				−3.29		−3.27	−1.30	−5.39										
WNbath				−1.55		−1.70	17.85 ***	−11.20 ***										
WPatio				−7.21 *		−7.28 *	14.31 ***	2.92										
WFirepl				10.96 *		10.75 *	31.64 ***	9.62 *										
WAC				17.45 ***		17.02 ***	26.05 ***	−1.10										
WBment				5.36		5.19	16.69 **	−0.82										
WNstor				0.35		0.27	8.19 ***	4.81										
WGar				7.69		7.59	0.71	0.22										
WAge				2.24		2.07	9.73 **	−0.02										
WCitcou				0.04		0.04	0.11	−6.87										
WLotsz				−1.71		−1.71	26.68 ***	0.07 *										
WSqft				0.10 **		0.10 **	0.14 ***	0.48										
rho		0.27 ***			0.36 ***	0.00		0.45 ***										
lambda			0.31 ***		−0.34		0.96 ***	−0.71 ***										
Log-Likelihood	−926.53	−817.7957	−751.92	−926.53	−816.07	−802.20	−778.74	−797.55				−908.65	−909.11	−920.57	−920.22	−921.09	−921.39	−920.79
Moran (*p*-value)	0.02	0.09	0.02	0.46	0.39	0.46	0.36	0.92	0.02	0.32	0.34	0.25	0.29	0.02	0.01	0.02	0.02	0.02
dh-test (*p*-value)	0.02	0.05	0.12	0.01	0.12	0.12	0.13	0.12	0.04	0.02	0.01	0.02	0.02	0.03	0.02	0.04	0.04	0.04
AIC	1.88	1.67	1.53	1.84	1.66	1.51	1.66	1.65		5.1	5.09	1.86	1.85	1.86	1.87	1.87	1.87	1.87
	OLS	SAR	SEM	SLX	SAC	SDM	SDEM	GNS	Spline	C-spline	F-spline	Model A	Model B	Model C	Model D	Model E	Model F	Model G

#of embedding dimensions m = 6; dh-test *p*-value on raw data 0.13; ***, **, * = coefficient estimates that are significant at the 0.01, 0.05 and 0.1 level, respectively.

**Table A2:** Results for Boston housing prices.

	OLS	SAR	SEM	SLX	SAC	SDM	SDEM	GNS	Spline	C-Spline	F-Spline	Model A	Model B	Model C	Model D	Model E	Model F	Model G
Constant	36.46 ***	19.25 ***	30.11 ***	37.77 ***	28.09 ***	23.94 ***	27.49 ***	25.74 ***		704,400.00 **	−274.30	46.69 ***	46.78 ***	34.64 ***	47.61 ***	47.57 ***	−435.14	−122.45
Crime	−0.11 ***	−0.09 ***	−0.13 ***	−0.12 ***	−0.13 ***	−0.12 ***	−0.13 ***	−0.15 ***	−0.15 ***	−0.12 ***	−0.12 ***	−0.13 ***	−0.13 ***	−0.11 ***	−0.13 ***	−0.13 ***	−0.11	−0.11 ***
Zoning	0.05 ***	0.04 ***	0.04 ***	0.03	0.04 ***	0.03 **	0.04 **	0.03 **	0.04 **	0.04 **	0.04 **	0.03 **	0.03 **	0.04 ***	0.03 **	0.03 **	0.05 ***	0.05 ***
Industry	0.02	0.02	−0.01	−0.01	−0.01	−0.05	−0.03	−0.02	−0.01	0.03	0.03	0.02	0.02	0.02	0.02	0.02	0.01	0.01
Charlesr	0.02 ***	1.29	−0.35	−0.54	−0.27	−1.31	−0.45	−0.1	1.33	2.42 **	2.15 *	2.68 ***	2.69 ***	2.54 ***	2.69 ***	2.67 ***	2.60 ***	2.59 ***
Noxsq	−17.77 ***	−13.06 ***	−18.58 ***	−29.57 ***	−17.80 ***	−17.96 ***	−18.72 ***	−19.23 ***	−23.05 ***	−20.18 ***	−19.33 ***	−21.80 ***	−21.83 ***	−16.01 ***	−22.92 ***	−23.09 ***	−15.90 ***	−15.90 ***
Rooms2	3.81 ***	3.68 ***	4.23 ***	4.06 ***	4.24 ***	4.33 ***	4.37 ***	4.66 ***	3.82 ***	3.62 ***	3.58 ***	3.74 ***	3.74 ***	3.78 ***	3.71 ***	3.71 ***	3.78 ***	3.78 ***
Houseage	0.01 ***	0.01	−0.02 *	−0.03 *	−0.02	−0.03 **	−0.03 **	−0.03 **	−0.03 *	−0.02	−0.02	−0.02	−0.02	0.00	−0.02	−0.02	0.01	0.01
Distance	−1.48 ***	−1.24 ***	−1.62 ***	−1.88 ***	−1.62 ***	−1.51 ***	−1.59 ***	−1.61 ***	−2.25 ***	−2.37 ***	−2.45 ***	−3.22 ***	−3.24 ***	−1.33 ***	−1.28 ***	−3.11 ***	−1.38 ***	−1.38 ***
Access	0.31 ***	0.26 ***	0.32 ***	0.35 ***	0.32 ***	0.29 ***	0.31 ***	0.31 ***	0.31 ***	0.36 ***	0.35 ***	0.36 ***	0.36 ***	0.33 ***	0.33 ***	0.34 ***	0.31 ***	0.31 ***
Taxrate	−0.01 ***	−0.01 ***	−0.01 ***	−0.01 ***	−0.01 ***	−0.01 ***	−0.01 ***	−0.01 ***	−0.01 ***	−0.01 ***	−0.01 ***	−0.01 ***	−0.01 ***	−0.01 ***	−0.01 ***	−0.01 ***	−0.01 ***	−0.01 ***
Pupil/Teacher	−0.95 ***	−0.63 ***	−0.64 ***	−0.61 ***	−0.64 ***	0.44 ***	−0.58 ***	−0.57 ***	−0.74 ***	−0.99 ***	−0.95 ***	−1.02 ***	−1.02 ***	−0.92 ***	−1.03 ***	−1.04 ***	−0.91 ***	−0.91 ***
Blackpop	0.01 ***	0.01 ***	0.01 ***	0.01 ***	0.01 ***	0.01 ***	0.01 ***	0.01 ***	0.01 ***	0.01 ***	0.01 ***	0.01 ***	0.01 ***	0.01 ***	0.01 ***	0.01 ***	0.01 ***	0.01 ***
Lowclass	−0.52 ***	−0.40 ***	−0.44 ***	−0.45 ***	−0.43 ***	−0.41 ***	−0.41 ***	−0.39 ***	−0.52 ***	−0.53 ***	−0.53 ***	−0.53 ***	−0.54 ***	−0.53 ***	−0.53 ***	−0.53 ***	−0.53 ***	−0.53 ***
WCrime				0.14 **		0.1	−0.05	−0.17 *										
WZoning				0.02		−0.01	0.01	0.02										
WIndustry				−0.04		0.03	0.05	0.05										
WCharlesr				7.91 ***		5.11 ***	7.09 ***	7.28 ***										
WNoxsq				11.65		5.79	−3.96	−11.02										
WRooms2				−0.76		−3.60 ***	0.53	2.89 *										
WHouseage				0.07 ***		0.06 **	0.04	0.02										
WDistance				0.72		0.94 *	0.12	−0.47										
WAccess				−0.25 *		−0.23 **	−0.06	0.07										
WTaxrate				0.01		0.01 **	0.01	0.01										
WPupil/Teacher				0.50 **		−0.13	−0.26	−0.25										
WBlackpop				−0.01		−0.01 **	−0.01	0.01										
WLowclass				−0.18 *		0.11	−0.1	−0.19										
rho		0.31 ***			0.06	0.59 ***		−0.31										
lambda			0.68 ***		0.64 ***		0.64 ***	0.81										
Log-likelihood	−1751.8	−1467.43	−1256.67	−1729.1	−1431.09	−1420.53	−1420.98	−1420.03				−1725.4	−1725.9	−1741.3	−1728.3	−1729.0	−1742.6	−1742.5
Moran (*p*-value)	0	0	0	0	0.46	0	0	0.54	0	0	0	0	0	0	0	0	0	0
dh-test (*p*-value)	0.04	0.10	0.12	0.05	0.01	0.07	0.09	0.01	0.09	0.03	0.03	0.03	0.03	0.05	0.05	0.04	0.04	0.04
AIC	3.52	2.96	2.54	3.47	2.89	2.53	2.88	2.88		3.1	3.09	3.49	3.49	3.52	3.52	3.52	3.52	3.52
	OLS	SAR	SEM	SLX	SAC	SDM	SDEM	GNS	Spline	C-spline	F-spline	Model A	Model B	Model C	Model D	Model E	Model F	Model G

#of embedding dimensions m = 7; dh-test *p*-value on raw data 0.20; ***, **, * = coefficient estimates that are significant at the 0.01, 0.05 and 0.1 level, respectively.

**Table A3:** Results for Toledo housing prices.

	OLS	SAR	SEM	SLX	SAC	SDM	SDEM	GNS	Spline	C-Spline	F-Spline	Model A	Model B	Model C	Model D	Model E	Model F	Model G
Constant	8105.30	8441.69	8410.68	−2831.41	8564.30	−4299.56	−7219.56	−3694.60		3.299 $\times \;10^{8}$	3.445 $\times \;10^{6}$ *	1,252,522,880	−685,092,529.5	1,090,229,886	1,821,513.36 **	1,834,964.09 **	3,664,887.02 **	1,224,987.63 **
Neighborhood	−2758.37	−2842.90	−2821.70	−1098.85	−2867.48	−895.53	−708.78	−882.77	−3696.39	−1348.00	−1162.00	−2165.26	−1886.25	−2935.71	−2583.57	−2377.45	−2377.25	−2377.64
Lot sqft	0.27	0.29	0.29	0.15	0.30	0.11	0.19	0.20	0.61	0.55	0.54	0.56	0.55	0.39	0.38	0.38	0.38	0.38
Total sqft	7.28	6.96	7.15	4.73	6.91	5.05	4.29	5.99	0.47	3.95	3.86	2.67	2.06	4.78	4.65	4.78	4.78	4.78
Family room	30,926.93 ***	30,859.95 ***	30,546.77 ***	21,272.60 **	30,718.27 ***	21,876.78 ***	20,990.69 **	19,292.71 **	23,652.30 **	29,010.00 **	28,620.00 **	25,736.93 ***	25,529.68 ***	30,432.49 ***	30,407.07 ***	30,322.9 ***	30,324.16 ***	30,321.64 ***
Rec room	−1608.90	−1860.70	−1832.99	−4541.93	−1944.34	−4104.28	−4953.70	5143.33	−6587.14	−4645.00	−5318.00	−5686.75 **	−5248.25 **	−2898.33 *	−2573.88 *	−2599.84 *	−2599.08 *	−2600.6 *
Air cond	−37,981.82 ***	−37,797.45 ***	−37,228.27 ***	−22,340.99	−37,519.17 ***	−23,296.76	−19,710.70	−18,300.67	−24,095.51	−27,840.00	−25,680.00	−22,861.58	−23,047.23	−34,972.25 **	−35,151.44 **	−34,790.75 **	−34,792.74 **	−34,788.76 **
Baths	16,449.63 ***	16,578.64 ***	16,604.19 ***	18,759.07 ***	16,616.57 ***	18,497.00 ***	18,988.56 ***	18,549.71 ***	20,972.00 ***	18,410.00 ***	18,650.00 ***	19,030.72 ***	19,253.35 ***	17,702.32 ***	17,747.41 ***	17,722.46 ***	17,721.84 ***	17,723.07 ***
Condition	8430.70 *	8766.38 **	8190.45 *	8004.52 *	8674.14 **	8672.42 **	8044.16 **	7720.24 *	4063.32	5434.00	4823.00	5442.27	5182.25	7984.58 *	7694.87	7471.27	7469.58	7472.95
Garage condition	−10,996.77 ***	−10,976.26 ***	−10,895.37 ***	−10,979.44 ***	−10,936.93 ***	−11,240.48 ***	−11,136.00 ***	−10,911.83 ***	−8194.50 ***	−9691.00 ***	−9479.00 ***	−9686.32 ***	−9809.15 ***	−10,572.82 ***	−10,628.51 ***	−10,584.73 ***	−10,584.11 ***	−10,585.34 ***
Age	−143.77	−149.68	−144.95	−223.14 **	−150.07	−225.93 **	−235.34 ***	−244.49 ***	−215.12 *	−257.00 *	−265.50 *	−261.17 **	−263.42 **	−175.77 *	−181.21 *	−188.06 *	−188.07 *	−188.05 *
Wneighborhood				3455.48		3092.83	2922.37	5347.99										
Wlotsqft				−1.72 *		−1.73 *	−1.72 **	−1.47 **										
Wtotalsqft				5.63		8.30	7.68	−2.88										
Wfamily room				20,387.20		24,679.64	17,493.16	15,391.37										
Wrecroom				14,095.51		14,814.49	15,395.43 *	15,748.86 **										
Wac				−52,454.49		−59,448.07	−50,695.33 *	−30,990.27										
Wbath				−4416.34		−2325.33	−5640.27	−3662.30										
Wcondition				12,393.72		15,397.47	13,039.72	7687.00										
Wgarcond				−3100.97		−5199.50	−2807.53	−3202.35										
Wage				−85.77		−120.96	−89.60	−18.34										
rho		−0.01			−0.02	−0.18		0.14										
lambda			0.03		0.01		−0.26	−0.40										
Log-likelihood	−1033.10	−989.26	−955.34	−1018.90	−989.25	−944.65	−978.24	−977.73				−1024.4	−1025.2	−1028.6	−1029	−1029.4	−1029.4	1029.4
Moran (*p*-value)	0.57	0.64	0.57	0.47	0.63	0.43	0.37	0.80	0.57	0.51	0.53	0.89	0.78	0.79	0.77	0.88	0.88	0.88
dh-test (*p*-value)	0.02	0.03	0.05	0.11	0.10	0.11	0.03	0.02	0.05	0.01	0.01	0.01	0.01	0.02	0.02	0.02	0.02	0.02
AIC	2.09	2.09	1.93	2.08	2.00	1.93	2.00	2.00		17.6	17.6	2.08	2.08	2.09	2.09	2.08	2.08	2.08
	OLS	SAR	SEM	SLX	SAC	SDM	SDEM	GNS	Spline	C-spline	F-spline	Model A	Model B	Model C	Model D	Model E	Model F	Model G

#of embedding dimensions m = 5; dh-test *p*-value on raw data 0.11; ***, **, * = coefficient estimates that are significant at the 0.01, 0.05 and 0.1 level, respectively.

## References

[1] Anselin L. (1988). Lagrange multiplier test diagnostics for spatial dependence and spatial heterogeneity. Geogr. Anal..

[2] Le Sage J.P. (2005). Spatial Econometrics. Encycl. Soc. Meas..

[3] Minguez R., Durban M.L., Basile R. (2019). Spatio-temporal autoregressive semiparametric model for the analysis of regional economic data. Stat. Methods Appl..

[4] Basile R., Minguez R. (2018). Advances in Spatial Econometrics: Parametric vs. Semiparametric Spatial Autoregressive Models. The Economy as a Complex Spatial System.

[5] McMillen D. (2012). Perspectives on spatial econometrcis: Linear smoothing with structured models. J. Reg. Sci..

[6] Gibbons S., Overman H.G. (2012). Mostly pointless spatial econometrics?. J. Reg. Sci..

[7] Mur J., Herrera M., Ruiz M. (2011). Selecting the W Matrix. Parametric vs. Nonparametric Approaches. MPRA Paper.

[8] Garcia-Cordoba J., Matilla-Garcia M., Ruiz Marin M. (2019). A test for deterministic dynamics in spatial processes. Spat. Econ. Anal..

[9] Caragliu A., Nijkamp P. (2015). Space and knowledge spillovers in european regions: The impact of different forms of proximity on spatial knowledge diffusion. J. Econ. Geogr..

[10] Basile R., Ciccarelli C. (2017). The location of the italian manufacturing industry, 1871–1911: A sectoral analysis. J. Econ. Geogr..

[11] Mur J., Trívez F.J. (2003). Unit roots and deterministic trends in spatial econometric models. Int. Reg. Sci. Rev..

[12] Anselin L., Lozano-Gracia N. (2009). Spatial Hedonic Models. Palgrave Handbook of Econometrics.

[13] Lopez F., Matilla-Garcia M., Mur J., Marin M.R. (2010). A non-parametric spatial independence test using symbolic entropy. Reg. Sci. Urban Econ..

[14] Ruiz M., Lopez F., Paez A. (2010). Testing for spatial association of qualitative data using symbolic dynamics. J. Geogr. Syst..

[15] Lopez F., Matilla-Garcia M., Mur J., Paez A., Ruiz M. (2016). A note on the sg (m) test. J. Geogr. Syst..

[16] Clapp J., Kim H.-J., Gelfand A. (2002). Predicting spatial patterns of house prices using LPR and Bayesian smoothing. Real Estate Econ..

[17] Day B., Bateman I., Lake I. (2007). Beyond implicit prices: Recovering theoretically consistent and transferable values for noise avoidance from a hedonic property price model. Environ. Resour. Econ..

[18] Chasco C., Le Gallo J., López F.A. (2018). A scan test for spatial groupwise heteroscedasticity in cross-sectional models with an application on houses prices in Madrid. Reg. Sci. Urban Econ..

[19] Dubin R.A. (1992). Spatial autocorrelation and neighborhood quality. Reg. Sci. Urban Econ..

[20] Pace R.K., Gilley O.W. (1997). Using the spatial configuration of the data to improve estimation. J. Real Estate Financ. Econ..

[21] Anselin L. (1996). Efficient Algorithms for Constructing Proper Higher Order Spatial Lag Operators. J. Reg. Sci..

